# Neuroimaging evidence of visual-vestibular interaction accounting for perceptual mislocalization induced by head rotation

**DOI:** 10.1117/1.NPh.11.1.015005

**Published:** 2024-01-31

**Authors:** Xin He, Min Bao

**Affiliations:** a Chinese Academy of Sciences, Institute of Psychology, CAS Key Laboratory of Behavioral Science, Beijing, China; b University of Chinese Academy of Sciences, Department of Psychology, Beijing, China; c State Key Laboratory of Brain and Cognitive Science, Beijing, China

**Keywords:** flash-lag effect, head rotation, visual-vestibular integration, hMT+, temporoparietal junction, functional near-infrared spectroscopy

## Abstract

**Significance:**

A fleeting flash aligned vertically with an object remaining stationary in the head-centered space would be perceived as lagging behind the object during the observer’s horizontal head rotation. This perceptual mislocalization is an illusion named head-rotation-induced flash-lag effect (hFLE). While many studies have investigated the neural mechanism of the classical visual FLE, the hFLE has been hardly investigated.

**Aim:**

We measured the cortical activity corresponding to the hFLE on participants experiencing passive head rotations using functional near-infrared spectroscopy.

**Approach:**

Participants were asked to judge the relative position of a flash to a fixed reference while being horizontally rotated or staying static in a swivel chair. Meanwhile, functional near-infrared spectroscopy signals were recorded in temporal-parietal areas. The flash duration was manipulated to provide control conditions.

**Results:**

Brain activity specific to the hFLE was found around the right middle/inferior temporal gyri, and bilateral supramarginal gyri and superior temporal gyri areas. The activation was positively correlated with the rotation velocity of the participant around the supramarginal gyrus and negatively related to the hFLE intensity around the middle temporal gyrus.

**Conclusions:**

These results suggest that the mechanism underlying the hFLE involves multiple aspects of visual-vestibular interactions including the processing of multisensory conflicts mediated by the temporoparietal junction and the modulation of vestibular signals on object position perception in the human middle temporal complex.

## 1 Introduction

Motion can distort the position perception of objects. Many mislocalizations are accompanied by existence of a moving visual stimulus.^1^^–^^3^ One of the best-known and most-studied motion-induced mislocalizations is the flash-lag effect (FLE),^4^^–^^6^ where a flash would be perceived as lagging behind a moving object even if they are actually aligned. In addition to the classical FLE, Schlag et al.^7^ found an extension of the FLE without stimulation of visual motion: a flash would be perceived as lagging behind an object that remains in one spot relative to the head when the observer is making head rotations. To account for this novel head-rotation-induced FLE (herein abbreviated as hFLE), Schlag et al. followed the “motion extrapolation” hypothesis proposed by Nijhawan^5^ that the neural system tends to predict the position of a moving object to compensate for the delay caused by the transmission and processing of neural signals. Although there was no retinal motion in this hFLE, Schlag et al. proposed that extraretinal motion could also be extrapolated.

As to the neural origin for the FLE, Maus et al.^8^ and Wang et al.^9^ proved the causal role of human middle temporal complex (hMT+) in FLE with TMS and tDCS, respectively. It is well known that hMT+ plays an essential role in forming the perception of visual motion and object position. This area not only represents the physical motion or position but also encodes implied motion, such as the motion aftereffect,^10^^–^^14^ and the perceived position of the observer.^15^^,^^16^ Thus, the hMT+ is involved in many mislocalizations induced by motion including the FLE.^17^^–^^19^ By contrast, to our knowledge, the brain activity in the hFLE has not been investigated. As a multisensory illusion, it can be foreseen that the hFLE involves even more complicated processing than its classical visual version, but still surprisingly little is known about it. Elucidating the mechanism underlying the hFLE may shed light on the complex visual-vestibular interactions that occur every day in natural viewing conditions. Furthermore, it will add to our knowledge of the vestibular cognition, which, differing from other sensory modalities, is extensively distributed across the brain with distinct physiological characteristics and awaits deeper explorations.^20^^–^^23^

In previous work, we pointed out that the motion extrapolation hypothesis could not provide an adequate explanation of the forming mechanism of hFLE.^24^ Instead, the account of visual-vestibular interaction was more preferred. We notice that vestibular stimulations^25^^–^^27^ and visual-vestibular integration^28^ can also activate hMT+. Moreover, hMT+ is functionally connected to several regions in the vestibular cortical network.^29^^,^^30^ Considering the contribution of hMT+ to the classical FLE and the functions of hMT+ concerning both motion processing and visual-vestibular integration, we hypothesized that hMT+ would be activated when observer experienced the hFLE.

To test this hypothesis, one should seek an appropriate neuroimaging tool first. Unfortunately, this is considerably challenging owing to the technical limitations for common tools (e.g., fMRI and EEG). Compared with fMRI and EEG that are both susceptible to motion artifacts, functional near-infrared spectroscopy (fNIRS) is more tolerant to motion and has thus been broadly employed in research on exercises, under natural scenes, or with special participants like infants. In this study, we measured with fNIRS the cortical activity around hMT+ of the participants who performed horizontal head rotations to experience the hFLE.

## 2 Methods

### 2.1 Participants

Twenty-eight participants (11 males and 17 females) were recruited for the research, whose ages ranged from 19 to 32 years (M±SD=25±3 years). The sample size was determined using G*Power^31^ after a pilot experiment with 11 participants. All the participants had normal or corrected-to-normal visual acuity and reported no history of motion sickness [including simulator sickness with VR or three-dimensional (3D) display] nor dysfunction of visual or vestibular system.

This study protocol was approved by the Institutional Review Board of the Institute of Psychology, Chinese Academy of Sciences. All the participants gave their informed consent and were paid after the experiment.

### 2.2 Apparatus

The experiment program was written and run with MATLAB R2013a (MathWorks Inc.) and Psychtoolbox v3.0.12^32^ on a Dell XPS 8700 PC (Dell Inc.), and the visual stimuli were presented to the participants on a Sony HMZ-T3 head-mounted goggle (Sony, Japan) with a field of view of 50 (horizontal) × 28 (vertical) degrees (deg) of visual angle, resolution of 1280×720  pixels, and refresh rate of 60 Hz. The goggles were worn outside the fNIRS headcap. Participants sat on a barber’s chair to achieve rotation with their feet off the ground, and a TSS-WL 3-Space wireless motion sensor (Yost Labs Inc.) was fixed to one arm of the chair.

The fNIRS recording was implemented by a LABNIRS continuous-wave fNIRS system (Shimadzu Inc., Japan) with the near-infrared wavelengths of 780, 805, and 830 mm. Optode localization was carried out with a Fastrak 3D tracking and digitizing system (Polhemus Inc.).

### 2.3 fNIRS Acquisition

**Fig. 1:**
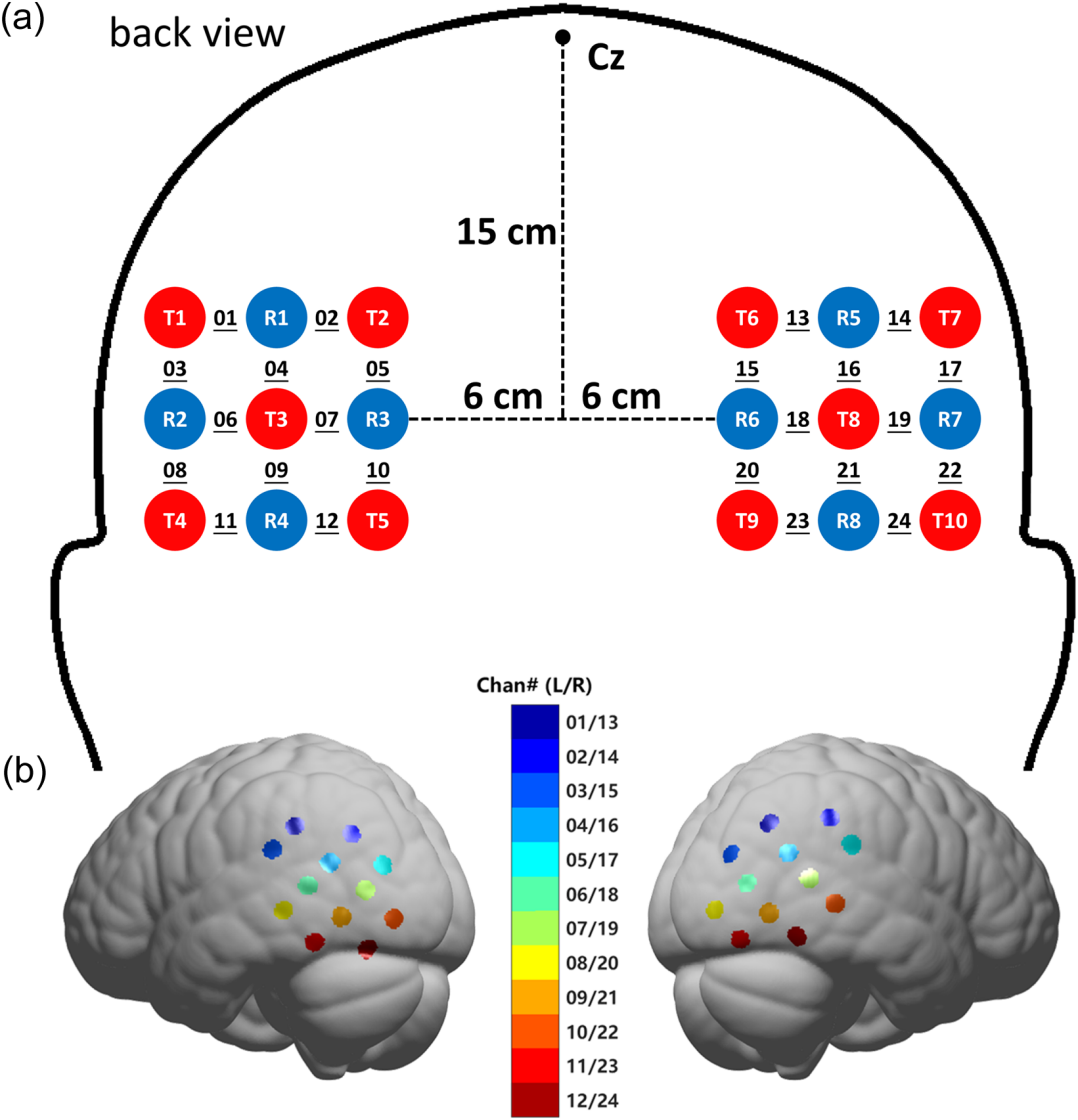
(a) The optode layout used in the study. Emitters are marked with red (T) and detectors with blue circles (R). Each adjacent pair of emitter and detector were 3 cm apart from each other to form a channel (underlined). There were 12 channels on each hemisphere and 24 in total. The distances marked are geodesic along the scalp. All the distances and sizes diagramed in the figure are for illustration purpose only and not in real scale. (b) Average locations of the channels on the brain template.

The optodes were arranged around T5/T6 of the international 10–20 system to cover bilateral temporo-parieto-occipital areas.^33^^–^^35^ On each side was a 3×3 square layout with five emitters and four detectors yielding 12 channels [Fig. 1(a)]. The raw sampling rate of fNIRS data was 55.6 Hz. After each participant completed the main task, a localization was carried out for each optode as well as for anatomical landmarks of the vertex (Cz), nasion (Nz), and bilateral preauricular points (AL/AR). These localization data were then used to estimate the locations of the channels on the Montreal Neurological Institute (MNI) brain template.^36^^–^^38^ The average locations of the channels on the brain template are shown in Fig. 1(b). The cortical subdivisions they fell on were then estimated based on the LONI Probabilistic Brain Atlas (LPBA40)^39^ and Brodmann areas (BA).

### 2.4 Stimuli and Procedure

We assumed that the relevant brain activation during the hFLE was the result of highly independent components caused by three types of neural signals, namely: (i) response to visual stimulus (i.e., the flash bar), (ii) response to vestibular stimulus (i.e., the horizontal body rotation), and (iii) the signal of visual–vestibular interaction leading to the hFLE. Based on this assumption, we designed five experimental conditions to decompose the brain activation during the hFLE.

#### 2.4.1 Short flash + moving (sF+M)

In light of previous findings where passive movement could also induce the hFLE,^40^ in this study, participants were seated in a swivel barber’s chair with their feet off the ground. The horizontal rotation was performed by the experimenter to reduce the influence of cervical movement on the fNIRS recording. The experimenter received a tone cue through earphones to start or stop the rotation. The display on the LCD monitor was identical to that on the goggles, allowing the experimenter to read the instruction.

Throughout the whole experimental session, the background of the display was pure black with a centered, red fixation point with 0.15 deg diameter. Before each block started, two lines of texts displayed the number of upcoming blocks and an instruction “Rotate!” to remind participants that they needed to rotate during the block. After participants were ready with their feet off the ground, the experimenter pressed a key to start the block. For the first 5 s, only a central reference bar and the fixation point were presented while a countdown with beep tones through earphones reminded the experimenter to prepare to rotate the chair. The reference bar was white with a size of 5 deg (height) × 0.5 deg (width).

**Fig. 2:**
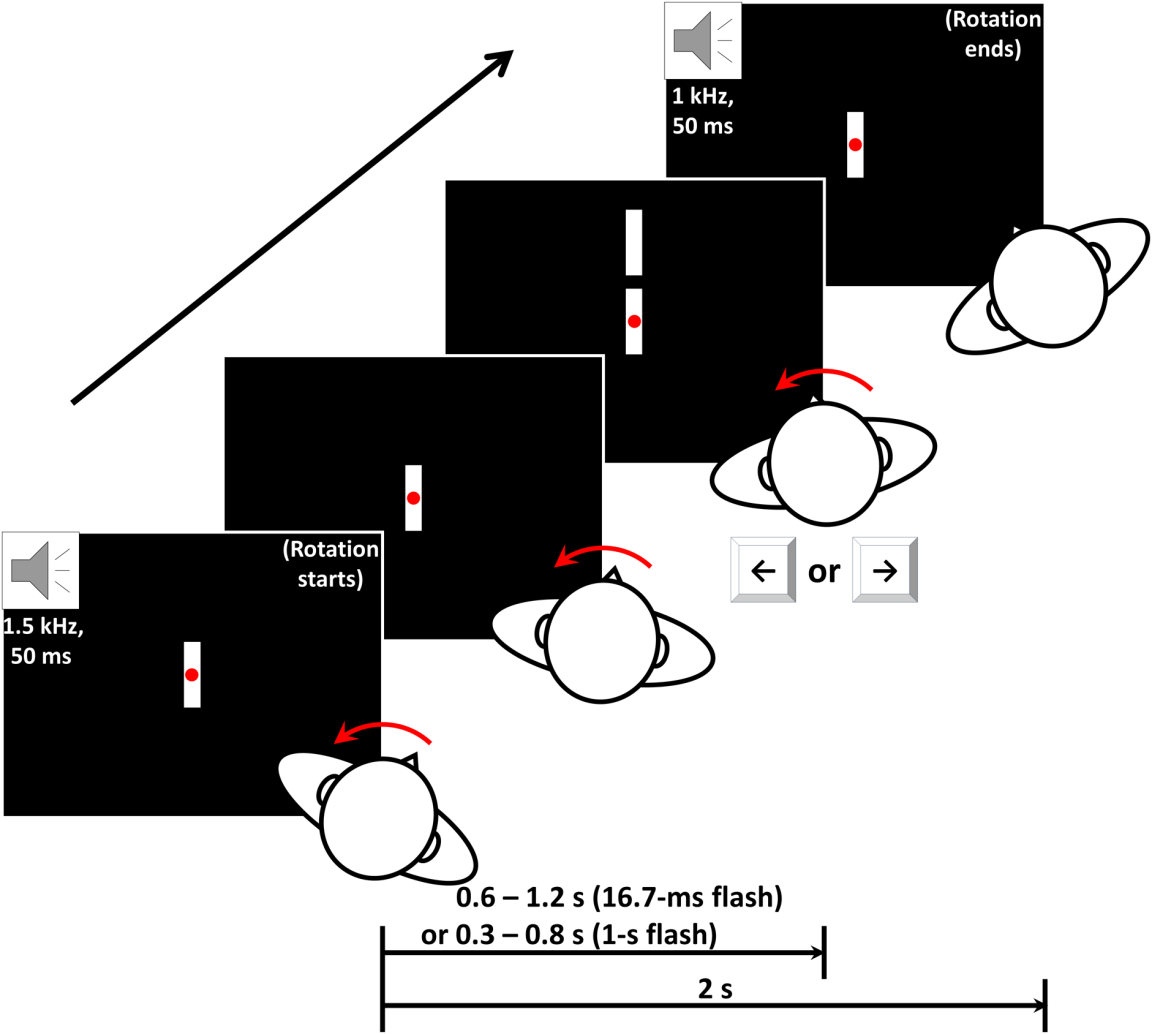
Illustration of a single trial in condition A or C. The experimenter started rotating the chair on hearing a high-pitch tone. 0.6 to 1.2 s (condition A) or 0.3 to 0.8 s (condition C) later, the flash bar appeared above the reference bar for 16.7 ms (condition A) or 1 s (condition C). The participant was asked to judge whether the flash bar was to the left or the right of the reference bar although the two bars were actually vertically aligned. The rotation was stopped after the low-pitch tone, which was 2 s later than the high-pitch tone. These auditory cues were played to the experimenter through earphones, so the participant could not hear them. The figure only showed the case where the participant was rotated leftward, but the rotating direction alternated between trials actually. All the distances and sizes of the visual stimuli in the figure are for illustration purpose only and not in real scale.

The experimenter rotated the chair once per trial based on the auditory cues from the earphones. A high tone (1500 Hz) indicated starting the rotation, and a low tone (1000 Hz) indicated ceasing, each lasting for 50 ms. Each rotation lasted for 2 s with 1-s intertrial intervals (Fig. 2). The experimenter purposely controlled the ends of rotation trajectories on approximately the same positions to keep the rotation amplitude roughly constant across trials. Each block consisted of 15 trials with alternating rotating directions between leftward and rightward.

After a random delay ranging from 0.6 to 1.2 s starting from the beginning of each rotation, a flash bar identical to the reference bar would appear 5 deg right above the reference bar for 1 frame (16.7 ms) and then vanish. Although the flash bar and target bar were vertically aligned physically, the participant was informed in advance that there was always a slight misalignment between the two bars and required to perform a 2AFC task discriminating whether the flash bar was to the left or the right of the reference bar by pressing the left or the right arrow key while maintaining fixation at the central fixation point. The reference bar remained centered on the display throughout each block.

The mean velocity across 0.5 s right before each flash onset was recorded as the rotation velocity. The average rotation velocities for conditions with rotation (i.e., conditions A, C, and E) are listed in Table 1.

**Table 1:** Summary of all conditions.

Signal	Condition				
	A (sF+M)	B (sF+S)	C (lF+M)	D (lF+S)	E (nF+M)
Visual	√	√	√	√	×
[duration (ms)]	[16.7]	[16.7]	[1000]	[1000]	—
Vestibular	√	×	√	×	√
[velocity (°/s)]	[42.11 ± 3.10]	—	[32.88 ± 2.77]	—	[41.81 ± 3.03]
hFLE (expected)	√	×	×	×	×

Note: Rotation velocities are reported in mean ± standard deviation. The velocity in condition C was lower than that in condition A because of their different flash onset delays relative to the beginning of trials (see Sec. 2.4).

#### 2.4.2 Short flash + static (sF+S)

Condition B was designed to measure the brain activation caused by the visual stimulus alone in condition A. Therefore, participants in condition B were not rotated. The other procedures and tasks were the same as those in condition A, except that the instruction before the block read “Do NOT rotate!” instead.

#### 2.4.3 Long flash + moving (lF+M)

Past studies have shown that the flash-lag effect may weaken with increasing duration of the flash stimulus.^41^^–^^44^ Furthermore, the upper limit of the flash duration that could induce the FLE was 80 to 500 ms.^41^^,^^42^^,^^44^^,^^45^ Thus, in condition C we lengthened the duration of the flash bar to 1 s. Hopefully, this modification might suppress the hFLE while maintaining similar visual stimuli to those in condition A. In terms of timing, the random delay before the flash was altered to 0.3 to 0.8 s (Fig. 2). Note that the rotation velocity recorded in condition C was lower than that in condition A (see Table 1) due to this shorter delay. The other procedures and tasks were identical to those in condition A, and participants were required to make responses after the flash bar disappeared.

#### 2.4.4 Long flash + static (lF+S)

Condition D was almost the same as condition C except that participants in this condition were not rotated. The instruction before the block also read “Do NOT rotate!” instead of “Rotate!”

#### 2.4.5 No flash + moving (nF+M)

Condition E served as a control to measure the brain activation caused solely by the self-rotation. Therefore, the flash bar was not presented and participants did not have to make any response. All the participant had to do was to keep gazing at the fixation point. The other procedures were the same as those in condition A. In particular, the program sent a trigger to the LABNIRS system to mark a virtual “flash-onset” event at a random time point of each trial to maintain a consistent data structure with the other conditions for the fNIRS data analyses.

**Fig. 3:**
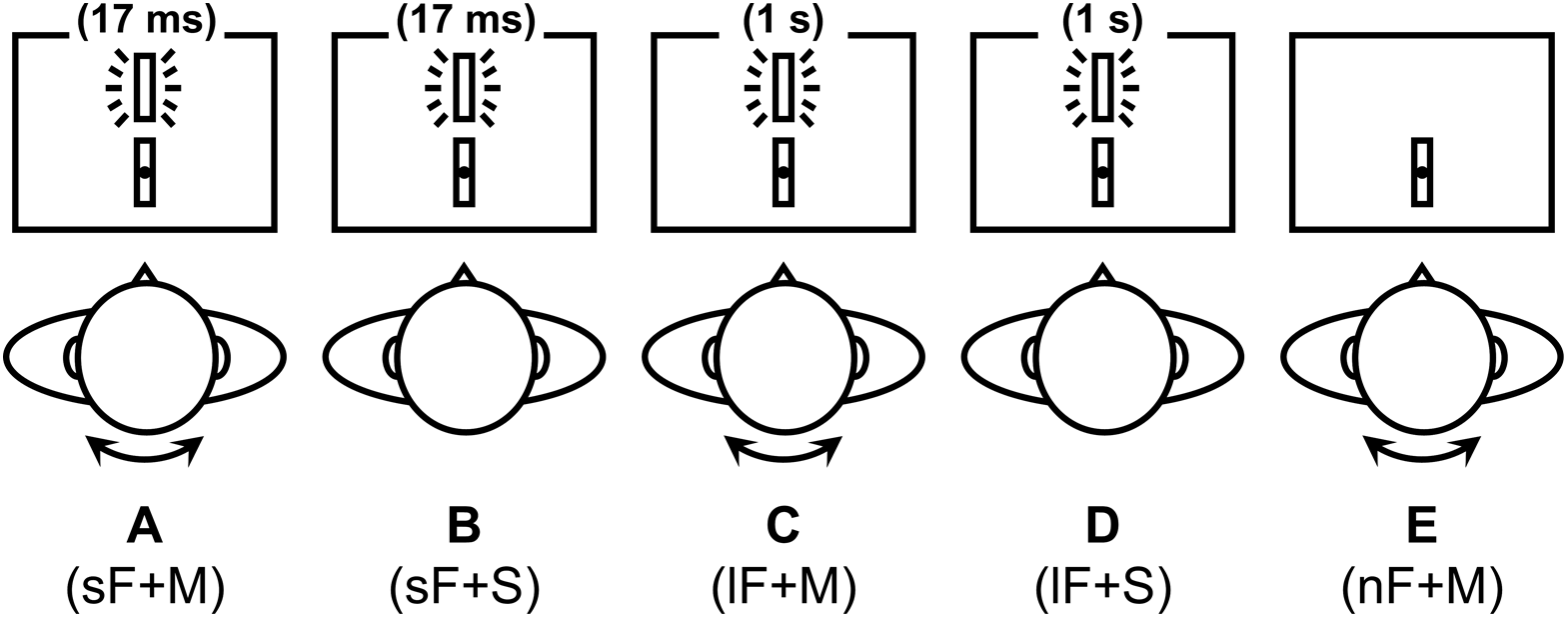
Simplified illustration and comparison of the conditions. The visual stimuli are for illustration only. For individual GLM-based analysis, a β-value for each condition was obtained ($\beta_{a}$ to $\beta_{e}$). Then, we examined the following three types of contrasts: (i) $\Delta \beta_{1}=(\beta_{a}-\beta_{b})-(\beta_{c}-\beta_{d})$, (ii) $\Delta \beta_{2}=(\beta_{a}-\beta_{b})-\beta_{e}$, and (iii) $\Delta \beta_{3}=(\beta_{c}-\beta_{d})-\beta_{e}$.

The conditions are summarized and compared in Fig. 3 and Table 1. Each condition consisted of five blocks, resulting in a total of 25 blocks for each participant, with the order randomized according to a Latin square design. Participants received a minimum of 15-s rest between blocks during which they could take a break without large head or body movements.

### 2.5 Behavioral Data Analysis

For conditions A and C, the proportion of responses that were in the opposite direction to self-rotation (e.g., reporting the flash bar to the left of the reference bar when rotating rightward and vice versa) was calculated to indicate the intensity of hFLE. If this proportion was significantly higher than 50%, it would indicate an hFLE. As for conditions B and D where the participants did not rotate, we simply calculated the proportion of responses reporting the flash bar to the left to measure response bias. A 2×2 repeated-measures ANOVA (short/long flash × moving/static) was then conducted on these response proportions to compare the intensity of hFLE across different conditions.

### 2.6 fNIRS Data Analysis

Both the absorbance (ABS) data of each wavelength and the relative changes in concentration of oxygenated, deoxygenated, and total hemoglobin (denoted as [HbO], [HbR], and [tHb], respectively) were obtained from the fNIRS system. These data were processed and analyzed using MATLAB 2017a (MathWorks Inc.) with the NIRS-KIT^46^ and SPM12^47^ packages.

#### 2.6.1 Pre-processing

**Fig. 4:**
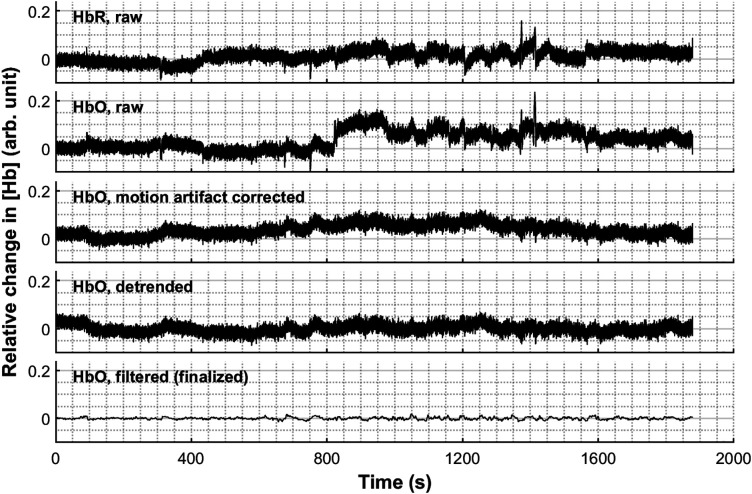
Demonstration of a sample of raw [HbO] time series (second row; downsampled already) undergoing the pre-processing procedure step by step (third to bottom row). This signal was obtained from Ch. #3 of participant #5. The corresponding raw [HbR] time series (top row) is also plotted as a comparison. All the signals are in arbitrary unit.

The pre-processing consisted of several steps. Figure 4 demonstrates the pre-processing pipeline step by step with an instance.

(I) Downsampling: We first downsampled the data from the sampling period of 18 to 54 ms (i.e., sampling rate from 55.56 to 18.52 Hz) using the resampling function built in the LABNIRS system. Then, both the ABS data and [Hb] data were exported as the raw data.
(II) Motion artifact correction: The head-motion-related artifacts were corrected with the temporal derivative distribution repair (TDDR) method^48^ built in the NIRS-KIT toolbox. Based on iteratively reweighting the temporal derivatives of the fNIRS signal, this parameter-free algorithm uses a robust regression approach to remove large fluctuations such as spikes and baseline shifts attributed to motion artifacts while leaving smaller, hemodynamic fluctuations, which can be used on either optical density or hemoglobin concentration signals.^46^^,^^48^^–^^51^
  To confirm the validity of the TDDR method, we conducted a repeated-measures ANOVA on the signal-to-noise ratio (SNR) of ABS data with three factors (wavelength, channel, and correction: raw versus TDDR-corrected). Focusing on the factor of correction, the results showed a significant main effect [F(1,27)=31.81, p<0.001, $\eta_{p}^{2}=0.54$], with an SNR increment (ΔSNR) of 3.42 dB after the TDDR correction. Also, the wavelength × correction interaction was significant [F(2,54)=8.31, p=0.001, $\eta_{p}^{2}=0.24$], showing that the correction effect varied across wavelengths but all valid ($\Delta {\mathrm{SNR}}_{780\;\mathrm{nm}}=3.61\;\mathrm{dB}$, $\Delta {\mathrm{SNR}}_{805\;\mathrm{nm}}=3.29\;\mathrm{dB}$, $\Delta {\mathrm{SNR}}_{830\;\mathrm{nm}}=3.34\;\mathrm{dB}$, ps<0.001). The channel × correction interaction was not significant [F(23,621)=1.17, p>0.3, $\eta_{p}^{2}=0.04$], indicating a relatively homogeneous correction effect across channels (ΔSNR range = [2.25, 5.70] dB, ps<0.014). The three-way interaction was not significant either [F(46,1242)=0.80, p>0.6, $\eta_{p}^{2}=0.03$]. These results proved that the TDDR method was suitable and effective for the current data.
(III) Detrending. The data were detrended using a second-order polynomial regression model.
(IV) Filtering. The data were filtered using a third-order IIR filter with a passband set at 0.01 to 0.39 Hz since the actual interstimulus intervals between flashes presented were all longer than 2.6 s.^52^
(V) Outlier rejection. The data at each channel of each participant were then segmented and analyzed block-wise, based on the time series from the first flash onset until the last flash offset of a block. The data were assessed with six indices: (1) SNR ($=20\;\log_{10}\;\frac{M}{\mathrm{SD}}$); (2) number of data loss (NDL), the number of the local data sequences with a fixed value (≥2 consecutive data points); (3) maximum continuous data loss (MCDL), the maximum length of the local data sequences with a fixed value; (4) repetition rate (RR), the proportion of repetitive values (= 1-number of distinct values/data length); (5) the range (R); and (6) the standard deviation (SD). Among them, the indices (2) to (6) were specially defined to automatically detect a certain type of low-quality data: upon visual inspection of the data, we noticed that many abnormal data involved a common pattern of varying between several limited values or remaining fixed for a period of time, which might not be screened by SNR because their SNR often seemed normal due to abnormally small SD. SNR was calculated for the average ABS of the three wavelengths, and the others for [HbO].
  First, SNR, NDL, and MCDL were calculated directly on the basis of raw data. Blocks with SNR<20  dB, NDL>6, or MCDL>2 were first rejected. Then, R, SD, and RR of the remaining blocks were assessed by their deviation within the distribution. Considering the high skewness of these distributions, we adopted the adjusted boxplot method^53^ with the LIBRA toolbox^54^^,^^55^ instead of the ordinary normalization. Blocks with R or SD beyond $\left[Q_{1}-1.5e^{-4\mathrm{MC}}\cdot \mathrm{IQR},Q_{3}+1.5e^{3\mathrm{MC}}\cdot \mathrm{IQR}\right]$ or RR beyond $\left[Q_{1}-30e^{-4\mathrm{MC}}\cdot \mathrm{IQR},Q_{3}+30e^{3\mathrm{MC}}\cdot \mathrm{IQR}\right]$ were further rejected ($Q_{1}$, first quartile; $Q_{3}$, third quartile; IQR, interquartile range; MC, medcouple; see Ref. 53). All the criteria except for SNR were determined to distinguish as many abnormal data described above from the rest as possible by visual inspection.
  Finally, only those channels that had all five conditions each containing at least one survival block could enter the further analyses. That is, if on a channel any condition had all its corresponding blocks rejected, then the whole channel would be excluded. Altogether, about 4.2% of all the blocks were rejected.

#### 2.6.2 First-level (individual) analysis

Analyses below mainly focused on the change of [HbO] as it is considered a more sensitive measure compared to [HbR] or [tHb]^56^^–^^58^ and many past studies have selected [HbO] as the main analysis target.^34^^,^^59^^–^^61^ However, the results for [HbR] will also be reported as a comparison.

The data were analyzed using a block design approach. The onset of the first flash and the offset of the last one in each block were defined as the beginning and end, respectively, and the duration between them was considered as the block duration. After pre-processed, the data of each participant were fitted to a GLM based on the canonical (two-gamma) hemodynamic response function provided by the SPM12 toolbox to obtain a β-value for each condition ($\beta_{a}$ to $\beta_{e}$). Then, we focused on three types of contrasts below: (i) $\Delta \beta_{1}=(\beta_{a}-\beta_{b})-(\beta_{c}-\beta_{d})$, (ii) $\Delta \beta_{2}=(\beta_{a}-\beta_{b})-\beta_{e}$, and (iii) $\Delta \beta_{3}=(\beta_{c}-\beta_{d})-\beta_{e}$.

According to the assumption mentioned above, $\beta_{a}$ contained all three types of signals while $\beta_{b}$ represented only the effect of the visual stimulus, so $\left(\beta_{a}-\beta_{b}\right)$ represented the vestibular component and the vestibular-visual interacting component (i.e., the hFLE). The case was similar for $\beta_{c}$ and $\beta_{d}$, except that the long flash was supposed not to induce the hFLE, so $\left(\beta_{c}-\beta_{d}\right)$ represented only the vestibular component. Therefore, $\Delta \beta_{1}$ should represent a relatively pure effect of the hFLE. We also calculated $\Delta \beta_{2}$ by replacing $\left(\beta_{c}-\beta_{d}\right)$ with $\beta_{e}$. And to examine whether the long flash was capable of inducing the hFLE, $\Delta \beta_{3}$ was also calculated.

#### 2.6.3 Second-level (group) analysis

We conducted one-sample t-tests to compare Δβ on each channel with a baseline of 0 in order to examine the differential brain activation across conditions. A significant deviation from 0 for $\Delta \beta_{1}$ or $\Delta \beta_{2}$ would indicate a potential locus that represented the hFLE on that channel. Besides, we analyzed the correlation between Δβ and rotation velocity or behavioral performance to relate neural activation to stimulus intensity or perception. As the analyses were conducted separately on each channel, multiple comparison corrections were implemented using the false discovery rate (FDR) method^62^ within either hemisphere.

For all the statistics above and below, effect sizes are reported as Cohen’s d (d for short) for t-tests or $\eta_{p}^{2}$ for ANOVA. All the p-values reported in the ANOVA have been adjusted where necessary using Greenhouse–Geisser correction, and post hoc tests were conducted with the Tukey–Kramer method.

## 3 Results

### 3.1 Behavioral Performance

**Fig. 5:**
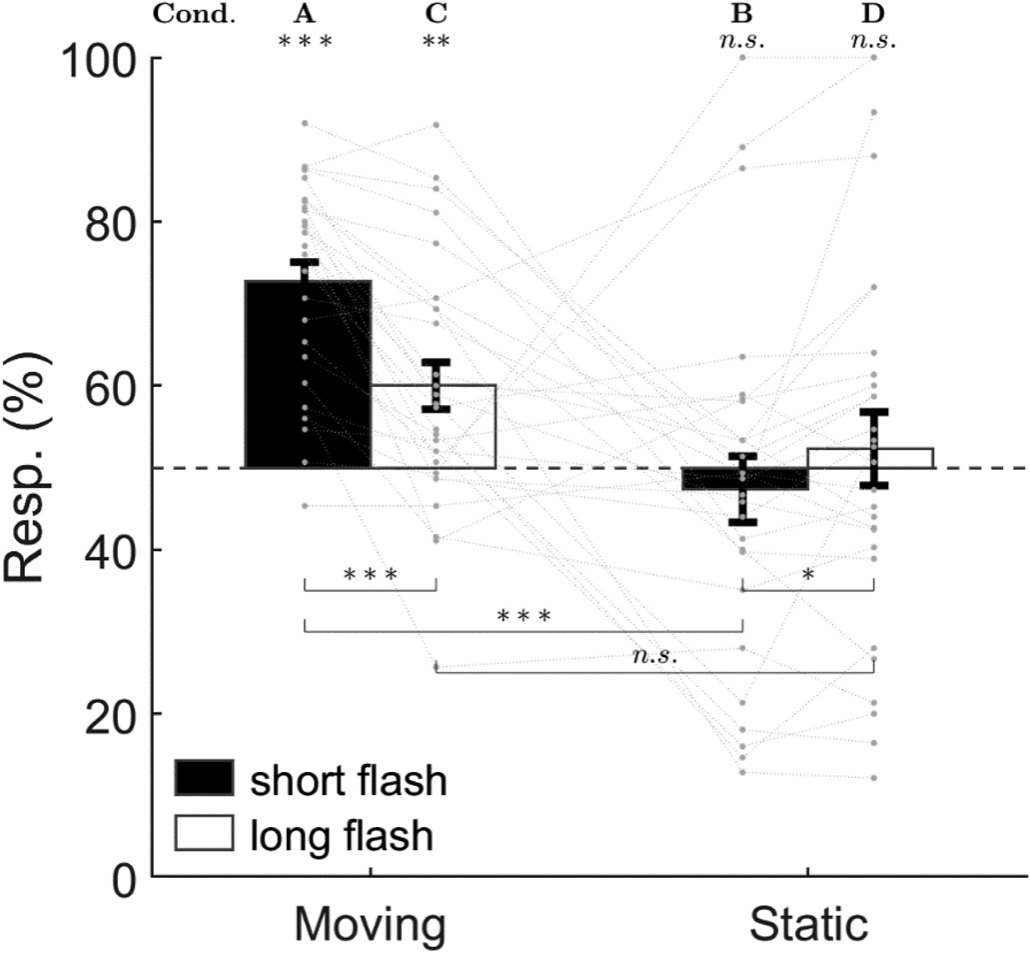
The behavioral results. Gray dots and dashed lines represent the individual data. Error bars indicate 1 SEM. n.s., not significant; * p<0.05; ** p<0.01; *** p<0.001.

According to the one-sample t-test results (Fig. 5), when the participants remained static (i.e., conditions B and D), their responses for the flash bar appearing on the left side did not deviate significantly from 50% [B: M±SD=(47.36±21.32)%; D: (52.31±23.93)%; ps>0.5], indicating little response bias. However, when the participants were moving (conditions A and C), responses for the flash bar appearing in the opposite side of the rotating direction were significantly greater than 50% of the total [A: (72.69±12.64)%, t(27)=9.50, p<0.001, d=1.80; C; (59.98±15.19)%, t(27)=3.48, p=0.002, d=0.66], which suggested an evident hFLE in both flash durations.

As for the ANOVA, the main effect of flash duration was significant [F(1,27)=9.68, p=0.004, $\eta_{p}^{2}=0.26$] with a higher response proportion for short flashes than for long ones, and so was the main effect of motion state [F(1,27)=12.01, p=0.002, $\eta_{p}^{2}=0.31$] with a higher response proportion when moving than when static. The interaction between flash duration and motion state was also significant [F(1,27)=22.33, p<0.001, $\eta_{p}^{2}=0.45$]: the response proportion was higher when moving than when static (p<0.001) but only for short flashes. In addition, the response proportion for short flashes was much higher than that for long flashes when moving (p<0.001) but slightly lower when static (p=0.043). These results showed that although either short or long flashes could induce the hFLE, the effect was relatively weaker for long flashes than for short flashes.

### 3.2 fNIRS

The actual sample sizes in the fNIRS analyses were less than the number of participants since some channels of some participants were excluded in the pre-processing stage, which will be reported below.

**Fig. 6:**
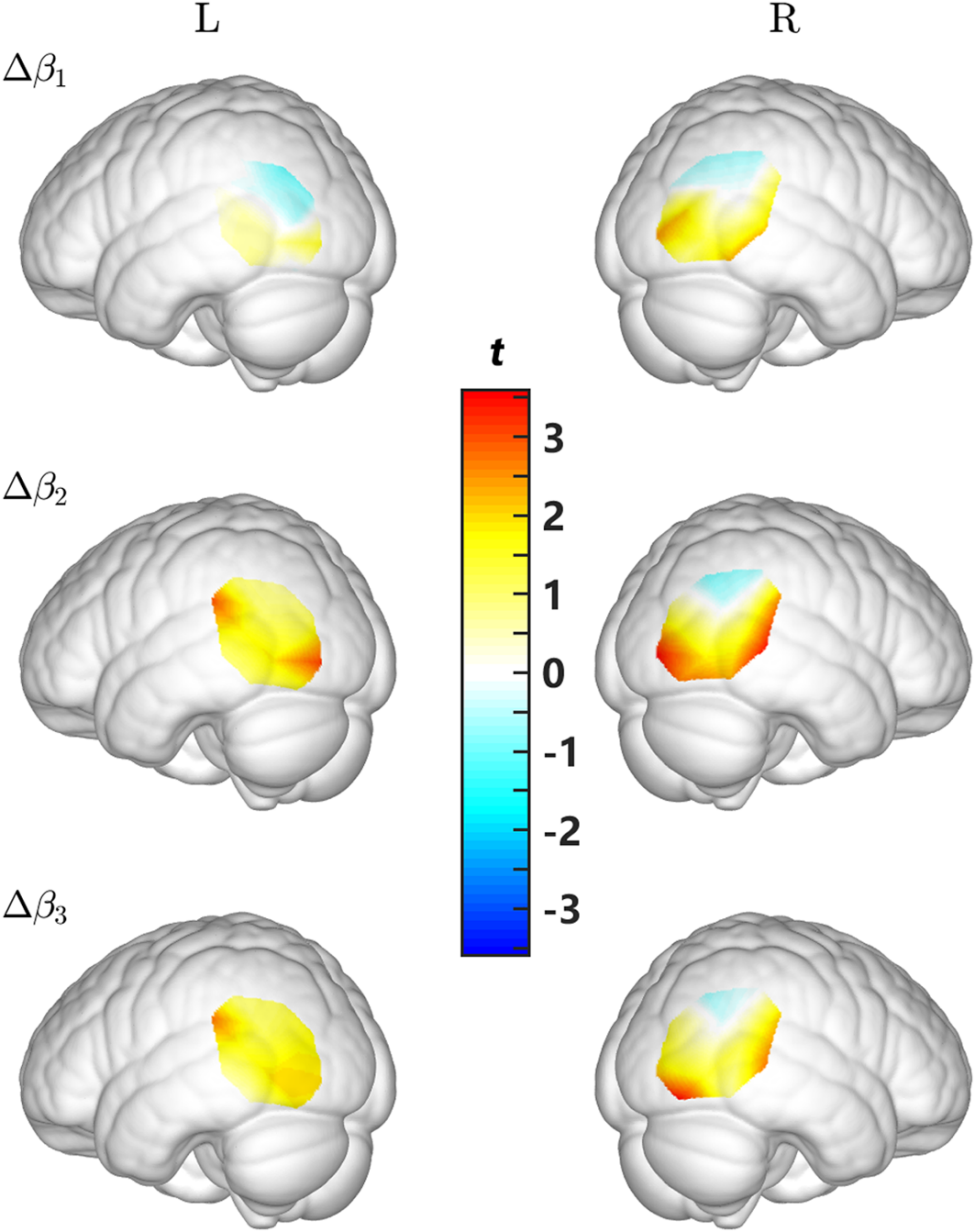
Results of t-tests (from top to bottom for $\Delta \beta_{1}$ to $\Delta \beta_{3}$, respectively).

First, we examined the deviations of Δβ from 0. For [HbO], as shown in Fig. 6 and Table 2, several channels showed significant deviation of $\Delta \beta_{1}$ from 0, but none of them survived the FDR correction. $\Delta \beta_{2}$ deviated significantly from 0 on more channels, among which Ch. #3, #10, #20, #22, and #23 remained significant after the FDR correction. Similarly, several channels showed significant $\Delta \beta_{3}$, and Ch. #23 passed the FDR correction. In contrast, for [HbR], Ch. #22 showed significant deviation of $\Delta \beta_{2}$ from 0 and #7, #14, and #22 showed significant deviation of $\Delta \beta_{3}$ from 0 (all ps<0.05), but none of them survived the FDR correction.

**Table 2:** Results of the examinations on Δβ with uncorrected ps<0.05.

	Ch. #	N	t	d
$\Delta \beta_{1}$	—	—	—	—
	18	28	2.08*	0.39
	20	28	2.46*	0.47
	22	28	2.18*	0.41
	24	27	2.33*	0.48
$\Delta \beta_{2}$	—	—	—	—
	3	26	**2.98** **	0.58
	10	25	**3.15** ***	0.63
	17	28	2.41*	0.46
	20	28	**3.57** ***	0.67
	22	28	**3.46** ***	0.65
	23	27	**2.73** *	0.52
	24	27	2.35*	0.45
$\Delta \beta_{3}$	—	—	—	—
	3	26	2.72*	0.53
	20	28	2.16*	0.41
	22	28	2.70*	0.51
	23	27	**3.59** ***	0.69

Note: N means the size of the sample actually included in the analysis on each channel, and bold font indicates surviving the FDR correction.

* p<0.05;

** p<0.01;

*** p<0.005

We then decided to focus on Ch. #3, #10, #20, and #22, which passed the FDR correction, as the main results. Although Ch. #23 also passed the FDR correction for $\Delta \beta_{2}$, it was excluded with caution because it did so for the control $\Delta \beta_{3}$ as well, which was not specific enough to the hFLE. Due to a similar reason, Ch. #17 and #24 were leniently selected because they showed relatively large raw $\Delta \beta_{2}$ but no $\Delta \beta_{3}$, which was consistent with the anticipation of the experiment. The results of localization for them are listed in Table 3.

**Table 3:** Brain regions corresponding to the focused channels.

Ch. #	MNI coordinates			Regions (probabilities)	
	x	y	z	LPBA40	BA
3	−68	−44	22	SMG (0.52)	22 (0.72)
				STG (0.47)	48 (0.24)
10	−43	−91	−7	MOG (0.77)	19 (0.63)
				IOG (0.23)	18 (0.37)
17	69	−43	25	STG (0.44)	22 (0.58)
				AG (0.31)	48 (0.21)
20	38	−96	−3	MOG (0.74)	18 (0.96)
				IOG (0.26)	19 (0.04)
22	69	−51	−2	MTG (0.92)	37 (0.53)
				ITG (0.08)	21 (0.34)
24	59	−66	−16	ITG (0.70)	37 (0.86)
				IOG (0.14)	19 (0.14)

Note: SMG, supramarginal gyrus; STG, superior temporal gyrus; MOG, middle occipital gyrus; IOG, inferior occipital gyrus; AG, angular gyrus; MTG, middle temporal gyrus; ITG, inferior temporal gyrus.

**Fig. 7:**
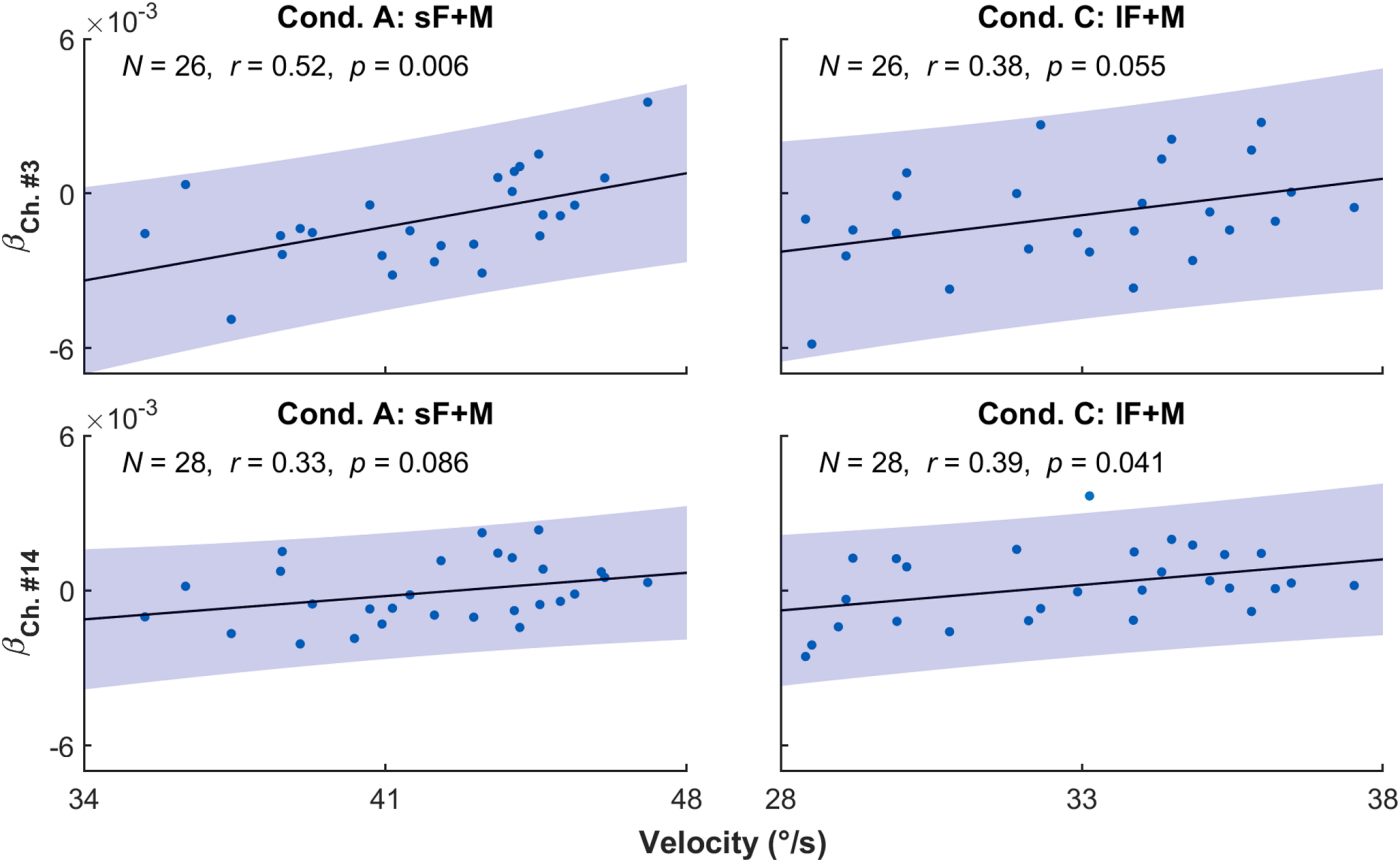
Correlation between β on Ch. #3 (upper row) and #14 (lower row) and rotation velocity in conditions A (left column) and C (right column).

**Fig. 8:**
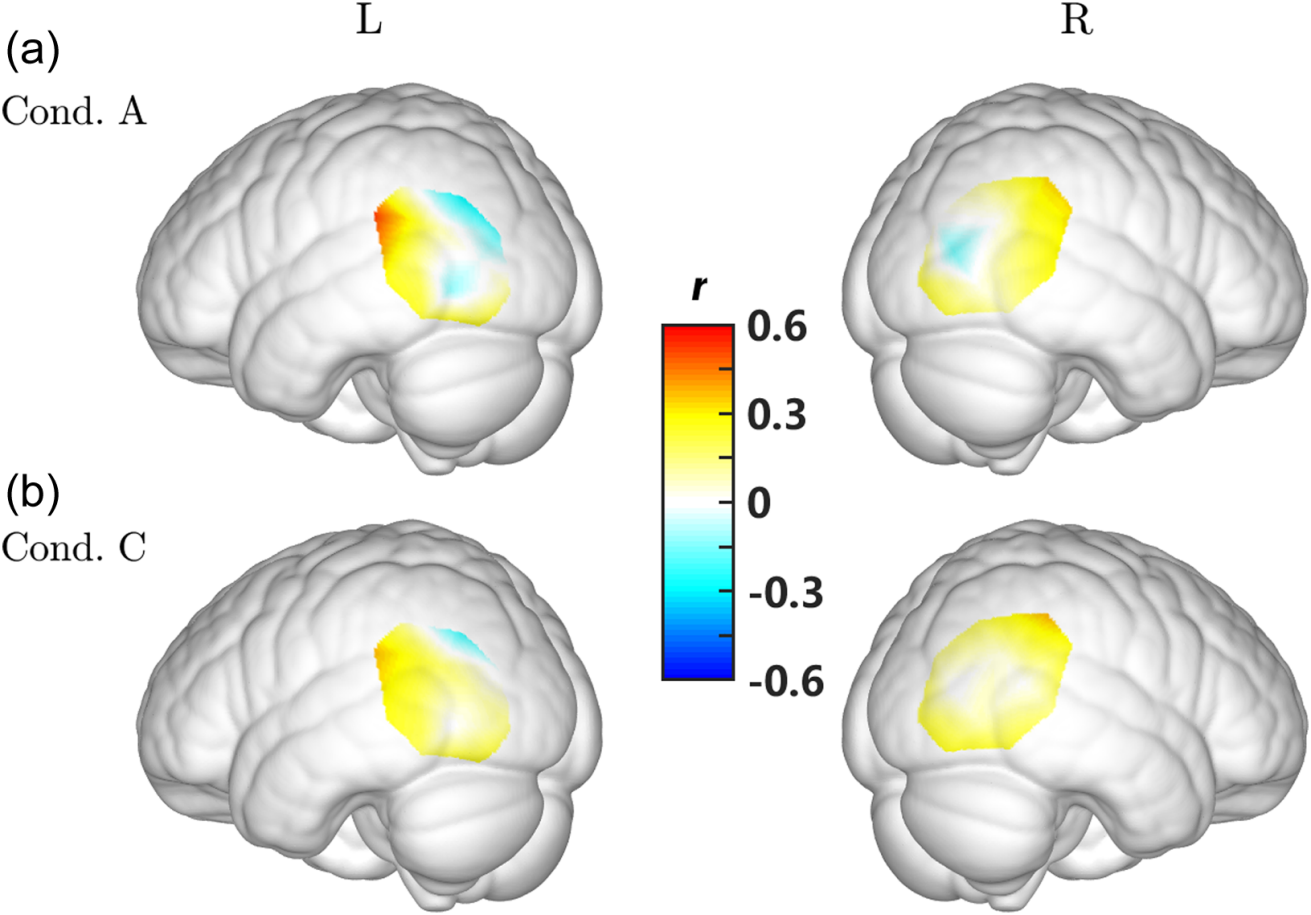
Distribution of the correlation between β and rotation velocity in conditions (a) A and (b) C.

The correlation between β and rotation velocity in the two conditions with rotation (i.e., A and C) was then explored. As shown in Fig. 7 and Table 4, Ch. #3 showed significant positive correlation in condition A (N=26, r=0.52, p=0.006) and Ch. #15 in condition C (N=28, r=0.39, p=0.041). Besides, both channels showed the trend of positive correlation on the other condition (Ch. #3, condition C: N=26, r=0.38, p=0.055; Ch. #15, condition A: N=28, r=0.33, p=0.086). In fact, the posterior temporal-parietal area was found to have a slightly higher correlation than other tested regions (Fig. 8).

**Table 4:** Channels with correlation to the rotation velocity.

Ch. #	MNI coordinates			Regions (probabilities)	
	x	y	z	LPBA40	BA
3	−68	−44	22	SMG (0.52)	22 (0.72)
				STG (0.47)	48 (0.24)
14	64	−52	37	AG (0.91)	40 (0.63)
				SMG (0.07)	39 (0.22)

Note: MTG, middle temporal gyrus; ITG, inferior temporal gyrus; AG, angular gyrus; SOG, superior occipital gyrus; MOG, middle occipital gyrus.

**Fig. 9:**
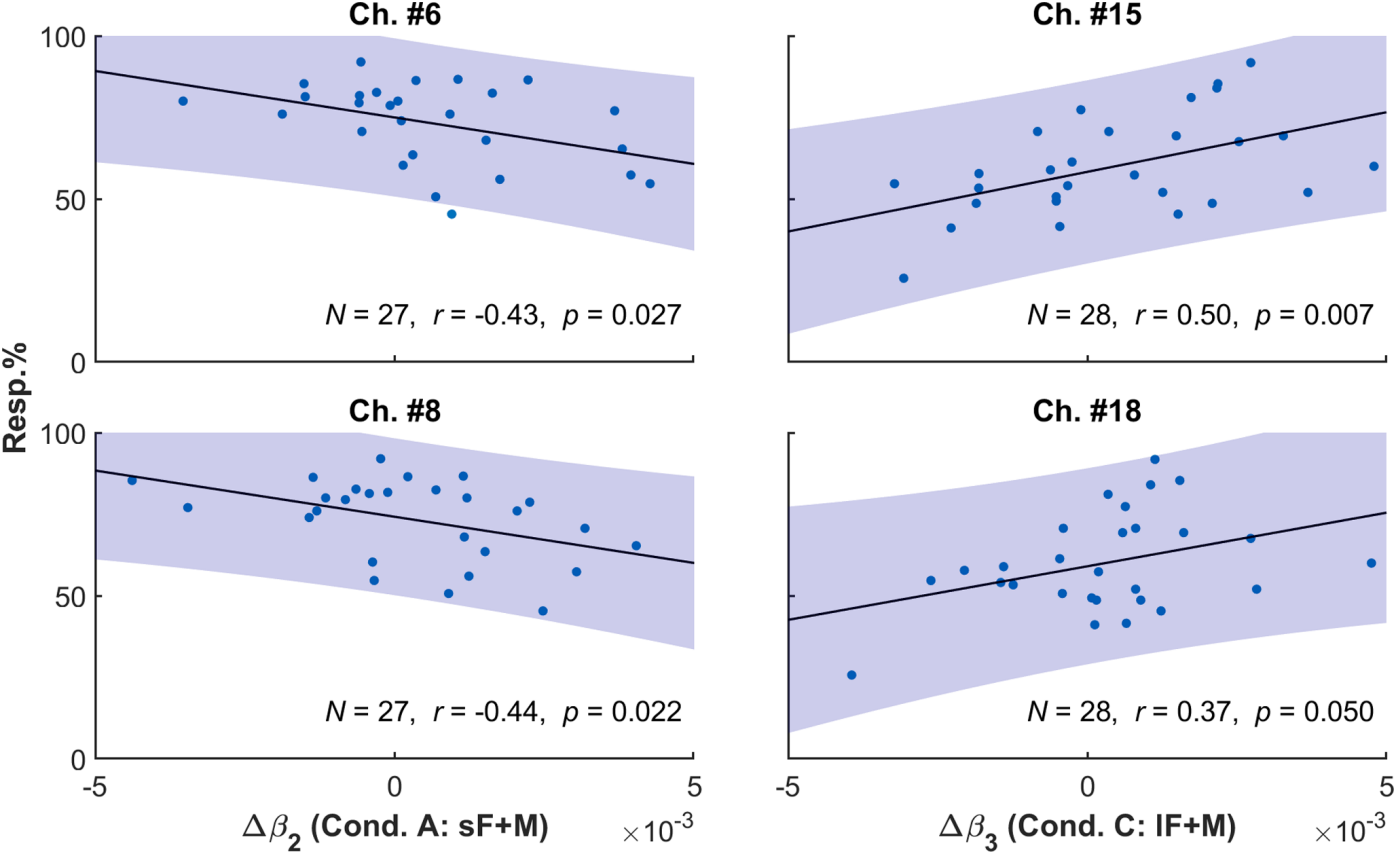
Correlation between Δβ and the corresponding response proportion.

**Fig. 10:**
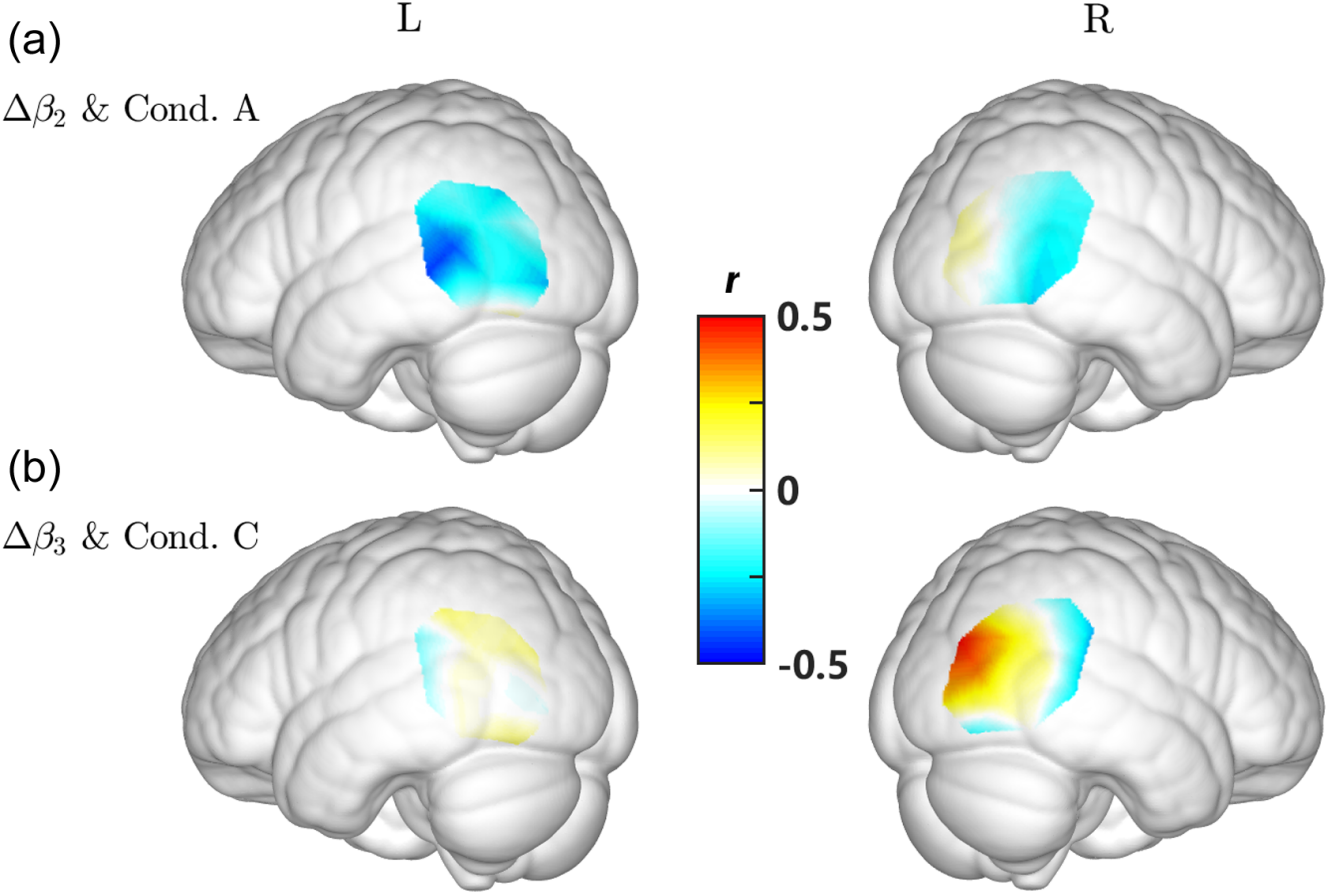
(a) Distribution of the correlation between $\Delta \beta_{2}$ and the behavioral performance in condition A and (b) between $\Delta \beta_{3}$ and the behavioral performance in condition C.

Finally, we tested the correlation between Δβ and the corresponding hFLE intensity in conditions A and C. None of the focused channels were found to have a significant correlation, but Ch. #6 (N=27, r=−0.43, p=0.027) and #8 (N=27, r=−0.44, p=0.022) showed significant negative correlation between $\Delta \beta_{2}$ and the response proportion in condition A, whereas Ch. #15 (N=28, r=0.50, p=0.007) and #18 (N=28, r=0.37, p=0.050) showed significant positive correlation between $\Delta \beta_{3}$ and the response proportion in condition C (Fig. 9). However, these results did not pass the FDR correction. The regions involved for these channels are listed in Table 5. As shown in Fig. 10, the negative correlation was mainly distributed across posterior part of the medium temporal gyrus in condition A, whereas the lateral middle occipital area showed positive correlation in condition C.

**Table 5:** Channels with correlation to the behavioral performance.

Ch. #	MNI coordinates			Regions (probabilities)	
	x	y	z	LPBA40	BA
6	−65	−59	6	MTG (0.95)	37 (0.77)
				AG (0.03)	21 (0.23)
8	−68	−49	−5	MTG (0.71)	37 (0.42)
				ITG (0.29)	21 (0.33)
15	40	−89	22	MOG (0.99)	19 (0.89)
				SOG (0.01)	18 (0.11)
18	49	−85	8	MOG (0.96)	19 (0.89)
				IOG (0.04)	18 (0.11)

Note: MTG, middle temporal gyrus; ITG, inferior temporal gyrus; AG, angular gyrus; SOG, superior occipital gyrus; MOG, middle occipital gyrus; IOG, inferior occipital gyrus.

However, no significant correlation was observed directly between rotation velocity and behavioral performance (condition A: r=−0.21, p>0.2; condition C: r=0.04, p>0.8).

In summary, the brain activity related to the hFLE was significant in right MTG/ITG (BA 37; Ch. #20 & #24), bilateral SMG/STG (BA 22; Ch. #3 & #17), and bilateral MOG/IOG (BA 18/19; Ch. #10 & #20). The activity in the SMG correlated with the rotation velocity, whereas that in the MTG correlated with the hFLE intensity.

## 4 Discussion

By using fNIRS, this study measured for the first time the brain activity corresponding to the hFLE. We provided the neuroimaging evidence for the engagement of left supramarginal gyrus (SMG)/superior temporal gyrus (STG) area (BA 22), the posterior part of bilateral middle temporal gyri (MTG)/inferior temporal gyri (ITG) areas (BA 37), and lateral occipital areas (BA 18/19).

To manipulate the hFLE while minimizing the alteration of the visual stimuli, we extended the duration of the flash bar based on previous literature on classical FLE.^41^^,^^42^^,^^44^ However, it turned out that the hFLE was reduced but not fully eliminated when the flash duration was extended to 1 s, as shown by the behavioral results that the hFLE for the long flash was also significant though weaker than that for the short flash and not significantly different from the control condition when the participants were static. Although it is difficult to propose an explicit account for this finding with only current data, we believe that it might reflect a certain difference between the mechanisms for the hFLE and classical FLE, which requires further empirical evidence. For example, the formation of hFLE might involve higher-level sensory processing such as multisensory interaction compared to the classical FLE and thus took a longer time span, which could cover a longer flash stimulus. On the other hand, the current behavioral task forced participants to discriminate the mislocalization between two physically aligned bars, resulting in virtually no signal to detect. Therefore, intrinsic neural noise might have a significant impact on perception,^63^^,^^64^ leading to the noticeable individual response biases in both static control conditions (B and D). This could also explain the observed hFLE for the long flash.

Consistent with our hypothesis that the hMT+ activates during the hFLE, Ch. #22 and #24 were identified by the analyses of Δβ contrasts of interest. Both channels are located in the middle and inferior temporal gyri (MTG and ITG) and both in BA 37. Despite that we did not functionally localize the exact divisions, we could infer from these estimated locations that they are likely situated in the middle temporal (MT) area, which is a main subregion of the hMT+, typically located around the occipital continuation and ascending branch of the inferior temporal sulcus.^65^^–^^68^ The hMT+ and its homolog in the macaque have been well recognized as key areas for processing visual motion information, both in macaques and humans. Interestingly, recent studies have demonstrated that they also to respond to vestibular stimuli and participate in visual-vestibular integration to form heading and self-motion perception.^25^^–^^27^^,^^69^^–^^74^ Della-Justina et al.^28^ presented participants with visual stimulus of flickering checkboards, vestibular stimulus of galvanic vestibular stimulation (GVS), and combined stimulus, and observed activation of ITG to single visual stimulus and visual-vestibular combined stimulus. Furthermore, Indovina et al.^30^ used graph-theoretical network analysis to find that BA 37 was structurally connected to the posterior insula cortex (PIC), a key hub in the vestibular cortical network, and that BA 37 itself also showed some hubness in the vestibular cross-modal network.

Based on these previous findings, we reckon that the activity of BA 37 is related to visual-vestibular integration processing that occurs in hMT+. The negative correlation between the activity in BA 37 and the behavioral performance may help clarify its role. Given that there was no visual motion stimulus in this study, it is likely that the activity of hMT+ represented the afferent retinal motion signals generated by eye movements and intrinsic neural noises of the participant. One possible explanation for this is that weaker activation of the hMT+ in a participant reflects fewer eye movements or neural intrinsic noises, resulting in weaker feedforward visual signals. This would lead to less weight of visual signals in the visual-vestibular integration and ultimately leave the estimation of object position more susceptible to the modulation of vestibular signals. Alternatively, the hMT+ could be subject to the inhibition by vestibular signals or by the feedback from visual-vestibular integration. Indeed, several past studies have observed mutual inhibition in visual-vestibular integration,^29^^,^^75^^,^^76^ especially when the stimulus from the suppressed modality was weak. This suppression might aim to reduce neural noise from the weak modality and resolve sensory conflict.^75^ In any case, the intensity of hFLE reflects the relative disadvantage of the visual modality compared to the vestibular modality in their integration during self-motion in our study.

On the other hand, Ch. #3 and #17 (and #14 in the correlation analysis) are located around SMG and STG (especially the posterior part, BA 22), two areas that also participate in visual-vestibular integration, especially the representation and processing of sensory conflicts.^27^^,^^33^^,^^77^ Both areas are parts of the temporoparietal junction (TPJ), a functional complex between the parietal and temporal lobes and around the posterior end of the Sylvian fissure that plays a critical role in human vestibular system (see Ref. 78 for review) and found to be functionally connected to PIC and the parietal-insular vestibular cortex, two essential hubs in the vestibular network.^30^^,^^79^^–^^81^ Previous neuroimaging research studies have found that the temporoparietal areas, including the SMG and STG, respond to caloric vestibular stimulation or GVS^22^^,^^82^ and activate in body-related tasks such as standing balance or inner verticality where the cues from other sensory modalities than vestibular were deprived,^83^^,^^84^ and the STG response increased with the GVS intensity.^82^ In an fNIRS study using horizontal rotation as the vestibular stimulation, Nguyen et al.^33^ found that TPJ was sensitive to the incongruency between visual and vestibular stimuli, and that the activation of dorsal SMG negatively correlated with the intensity of subjective vertigo reported by the participant.

Although both hMT+ and TPJ engage in visual-vestibular integration, there are still some differences. Compared with hMT+, which is more of a visual related area, TPJ is closer to vestibular processing in visual-vestibular integration. This could also explain the different correlation results. The brain activation to the hFLE on Ch. #3 and #14 (TPJ) was positively correlated to the velocity probably because TPJ was more active in solving bigger visual-vestibular conflicts when the vestibular stimulation was stronger. In contrast, the brain activity on Ch. #6 and #8 identified in the correlation analysis on β and located in hMT+ influenced behavioral responses, which reflected the visual perception and subsequent sensory decision making affected by the visual-vestibular interaction.

As to Ch. #10 and #20 (and #15 and #18 in the correlation analysis), they fell in the lateral part of the medial occipital area (BA 18/19, extrastriate visual cortex V2/V3). The brain activity here might be reflective of the remaining effect from the visual stimulus of the flash, which was not fully eliminated in $\Delta \beta_{2}$ and $\Delta \beta_{3}$. This also explains the two distinct types of correlations between Δβ and the hFLE intensity in conditions A and C: in condition A with a weak visual input and strong hFLE, the performance was mainly determined by the cross-modal interaction in hMT+ as stated above, whereas in condition C where the visual input was relatively strong and the illusion was weak, the perception was likely influenced much by the response bias, which reflected the intrinsic neural noise of the extrastriate visual cortex V2/V3, as discussed at the beginning of the section.

Finally, despite that the brain activity was observed to correlate with both the rotation velocity and the hFLE intensity, no significant correlation was found directly between the latter two, which was consistent with our previous findings.^24^ In that study, an hFLE was significantly induced both when the participants actively rotated the head (experiment 1, HM condition) and when they were passively rotated in the chair by the experimenter (experiment 1, BM condition), but the hFLE intensity significantly correlated to the rotation velocity only in the HM and not in the BM condition, which is interesting especially because these two conditions did not show significant difference in the hFLE intensity. And that finding in the BM condition was replicated in this study. We suspect that the decisive process to cause hFLE depends solely on the vestibular stimulation and does not distinguish its source, i.e., whether it comes from an active or a passive self-motion, but the intensity of the hFLE might be modulated by some mechanisms regarding this source, such as corollary discharge.^85^^–^^87^

Note that our findings were not symmetric across both hemispheres, with activation in the right hemisphere being generally stronger. This might be attributed to the fluctuations and noise in neural activity and data acquisition, but another possibility could be lateralization. Many studies have shown that at least part of the vestibular network was lateralized both anatomically and functionally toward a right-sided dominance,^23^^,^^29^^,^^30^^,^^88^^–^^90^ including when processing horizontal rotating directions.^91^ This lateral dominance was proved dependent partly on the handedness and often showed in the non-dominant hemisphere,^81^^,^^92^ which might account for the current findings, but other factors and mechanisms are also involved.^22^^,^^30^^,^^88^^,^^93^

An important concern awaiting future investigations is to detail the distinct division of labor in the contribution of these brain regions to the hFLE as well as other visual-vestibular interaction instances, considering the widespread distribution of the vestibular network across the cortex.^21^^,^^23^^,^^94^^–^^96^ These different roles may even relate to unique physiological characteristics such as elongated hemodynamics response.^22^ And the current results should be interpreted cautiously and further evidence is still needed due to several limitations. First, the spatial resolution of fNIRS is much lower than fMRI, and fNIRS can only detect cortical surface activation due to its imaging principle. Therefore, the results may not be sufficiently accurate or complete. Besides, the brain atlas used here is mainly anatomical rather than functional, making it challenging to match the current findings to past fMRI research about visual-vestibular integration. Future studies can use neuronavigation techniques to help access more precise ROIs and improve data reliability^97^^–^^99^ and neuromodulation tools such as TMS to confirm whether these areas play a causal role. Also, a motion platform^100^ can be used to implement finer and stricter manipulations of the motion profiles.

## 5 Conclusion

This study observed the activation of right SMG/STG area (BA 22), bilateral MTG/ITG areas (BA 37), and bilateral MOG/IOG areas (BA 18/19) in participants experiencing the hFLE. The activation of SMG was positively correlated with the rotation velocity while the activation of MTG was negatively correlated with the intensity of the hFLE. These findings demonstrate the role of visual-vestibular interaction in the formation of hFLE and provide indirect support to the visual-vestibular interaction account of the hFLE,^24^ suggesting that multiple visual-vestibular interactions help form the hFLE including the processing of multisensory conflicts in TPJ and the biasing of position perception by vestibular information in hMT+. Another minor but intriguing observation was that the hFLE could tolerate a longer duration of the flash stimulus compared to the classical visual FLE, indicating distinct mechanisms of visual processing between these two phenomena. This study also lends more support to the application of fNIRS in multisensory neuroimaging investigations that allows vestibular stimulations of real motion.

## Data Availability

The data that support the findings of this study are available from the corresponding author upon reasonable request.
